# Crystal structure of bis­(2-{[1,1-bis­(hy­droxy­meth­yl)-2-oxidoeth­yl]imino­meth­yl}-6-meth­oxy­phenolato)manganese(IV) 0.39-hydrate

**DOI:** 10.1107/S2056989015018551

**Published:** 2015-10-10

**Authors:** Elena A. Buvaylo, Olga Yu. Vassilyeva, Brian W. Skelton

**Affiliations:** a Department of Chemistry, Taras Shevchenko National University of Kyiv, 64/13 Volodymyrska Street, Kyiv 01601, Ukraine; b Centre for Microscopy, Characterisation and Analysis, M313, University of Western Australia, Perth, WA 6009, Australia

**Keywords:** crystal structure, monomeric octa­hedral Mn^IV^ complex, Schiff base ligand, *o*-vanillin, tris­(hy­droxy­meth­yl)amino­methane

## Abstract

The title compound, [Mn(C_12_H_15_NO_5_)_2_]·0.39H_2_O, is a 0.39 hydrate of the isostructural complex bis­(2-{[1,1-bis­(hy­droxy­meth­yl)-2-oxidoeth­yl]imino­meth­yl}-6-meth­oxy­phenolato)manganese(IV) that has previously been reported by Back, Oliveira, Canabarro & Iglesias [*Z. Anorg. Allg. Chem.* (2015), **641**, 941–947], based on room-temperature data. The current structure that was determined at 100 K reveals a lengthening of the *c* cell parameter compared with the published one due to the incorporation of the partial occupancy water mol­ecule. The title compound crystallizes in the tetra­gonal chiral space group *P*4_1_2_1_2; the neutral [Mn^IV^(C_12_H_15_NO_5_)_2_] mol­ecule is situated on a crystallographic *C*_2_ axis. The overall geometry about the central manganese ion is octa­hedral with an N_2_O_4_ core; each ligand acts as a meridional ONO donor. The coordination environment of Mn^IV^ at 100 K displays a difference in one of the two Mn–O bond lengths, compared with the room-temperature structure. In the crystal, the neutral mol­ecules are stacked in a helical fashion along the *c*-axis direction.

## Abstract 2

Using a predesigned tripodal Schiff-base ligand, a monomeric octa­hedral Mn^IV^ complex has been synthesized and its structure determined at 100 K.

## Chemical context   

The title compound is a hydrate of the isostructural complex bis­(2-{[1,1-bis­(hy­droxy­meth­yl)-2-oxidoeth­yl]imino­methyl}-6-meth­oxy­phenolato)manganese(IV) (refcode IGOSII; Back *et al.*, 2015 ▸). It was isolated as an unexpected product in an attempt to prepare a heterometallic Mn/Zn compound with the multidentate Schiff base ligand 2-{[(2-hy­droxy-3-methoxy­phen­yl)methyl­ene]amino}-2-(hy­droxy­meth­yl)-1,3-propane­diol (H_4_ *L*) (Odabaşoğlu *et al.*, 2003 ▸). Zn powder and MnCl_2_·4H_2_O were reacted with the Schiff base formed *in situ* from the condensation between *o*-vanillin and tris­(hy­droxy­meth­yl)amino­methane in methanol in a 1:1:2 molar ratio. Metal powders have been successfully applied in *direct synthesis of coordination compounds* to yield a number of novel monometallic (Babich & Kokozay, 1997 ▸; Babich *et al.*, 1996 ▸; Kovbasyuk *et al.*, 1997 ▸) and heterometallic complexes (Nikitina *et al.*, 2008 ▸; Nesterov *et al.*, 2011 ▸) of various nuclearities and dimensionalities. However, the isolated black microcrystalline product of the reaction studied appeared to be the mononuclear Schiff base complex [Mn^IV^(H_2_ *L*)_2_]·0.39H_2_O (**1**). Oxidation of the manganese(II) atom directly to the manganese(IV) species proceeds easily in open air even in the presence of zerovalent Zn, indicating that the tridentate ligand H_2_ *L* ^2–^ containing two O^−^ donors effectively stabilizes the Mn^IV^ oxidation state. Stabilization of Mn^IV^ species by similar ligands with phenolate oxygen atoms has been reported previously (Kessissoglou *et al.*, 1987 ▸; Pradeep *et al.*, 2004 ▸).
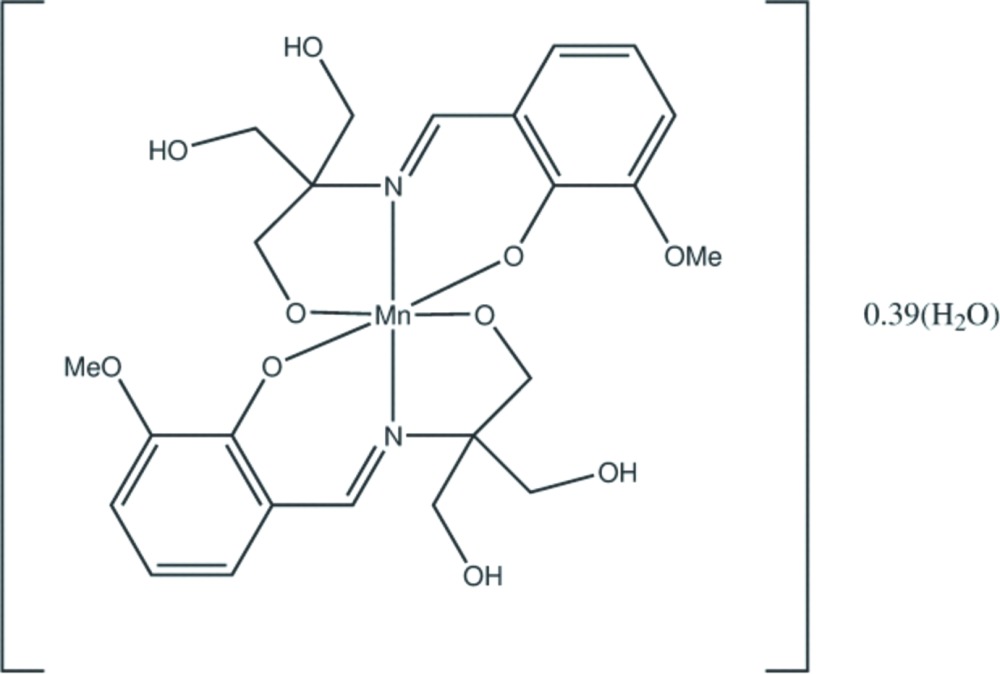


Remarkably, the current structure that was determined at 100 K reveals shortening of the *a* cell parameter compared with the published one [8.0953 (2) (**1**), 8.1620 (2) Å (IGOSII)] as expected in the case of low-temperature determination, but lengthening of the *c* cell parameter [37.568 (2) (**1**), 37.4557 (11) Å (IGOSII)] due to the incorporation of the partial occupancy water mol­ecule. Also, (**1**) shows somewhat longer Mn—O bond lengths to the deprotonated hy­droxy­methyl group [1.871 (4) Å] compared to the corresponding distance in IGOSII [1.849 (2) Å], while the Mn—N bonds stay the same [1.992 (5) (**1**), 1.991 (3) Å (IGOSII)].

## Structural commentary   

**Figure 1:**
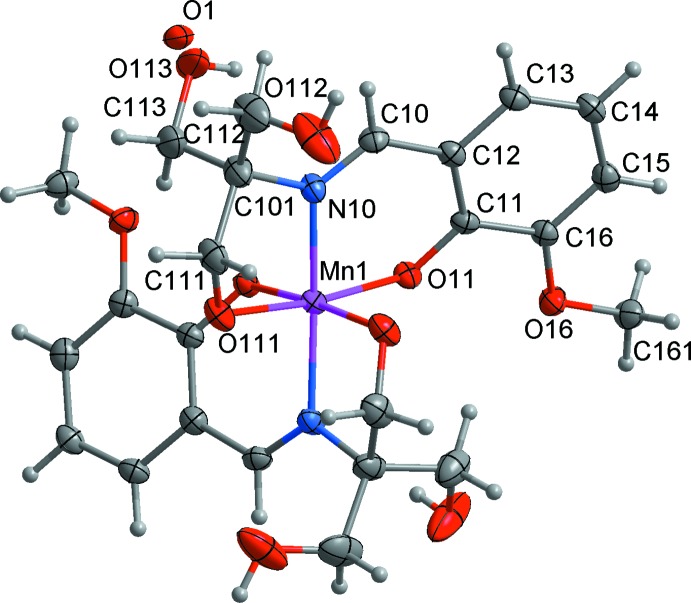
The mol­ecular structure of the title complex, showing the atom-numbering scheme. Non-H atoms are shown with displacement ellipsoids at the 50% probability level. Labelled atoms are related to unlabelled atoms by the symmetry operation *y*, *x*, −*z* + 1.

The title compound (**1**) crystallizes in the tetra­gonal chiral space group *P*4_1_2_1_2; the neutral [Mn^IV^(C_12_H_15_NO_5_)_2_] mol­ecule is situated on a crystallographic *C*_2_ axis, hence the asymmetric unit comprises one half of the metal complex and the O atom of a water mol­ecule with occupancy 0.195 (15) (Fig. 1 ▸). The overall geometry about the central metal ion is distorted octa­hedral with an N_2_O_4_ core; each ligand acts as a meridional ONO donor. The Mn^IV^—N(imine) [1.992 (5) Å], Mn^IV^–O(phenolate) [1.939 (4) Å] and Mn^IV^—O(alkoxo) [1.871 (4) Å] bond lengths in (**1**) are strictly comparable to those for several reported Mn^IV^ complexes containing similar ligation (Kessissoglou *et al.*, 1987 ▸; Pradeep *et al.*, 2004 ▸). The MnO_4_ equatorial fragment is approximately square planar, the maximum deviation from the mean plane being about 0.11 Å. The ranges of *cis* and *trans* angles at the metal atom are 84.14 (18)–98.44 (18) and 168.6 (3)–172.89 (18)°, respectively (Table 1 ▸). The Mn—N distance is longer than the average Mn—O distance by approximately 0.1 Å. This is significantly larger than the difference in covalent radii of N and O. Thus, the primary distortion of the MnN_2_O_4_ octa­hedron is axial elongation along the MnN_2_ axis.

**Figure 2:**
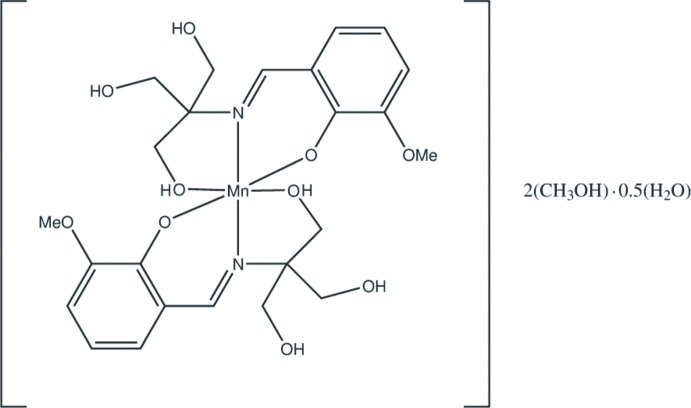
Scheme showing the structure of the closely related ROMROB Mn^II^ complex.

The mol­ecular structure of (**1**) closely resembles that of the Mn^II^ complex of the same ligand, [Mn^II^(H_3_ *L*)_2_]·2CH_3_OH·0.5H_2_O (refcode ROMROB; Zhang *et al.*, 2009 ▸) (Fig. 2 ▸). The latter crystallizes in the monoclinic space group *P*2_1_/*n* and has no crystallographically imposed symmetry. There is a marked increase in the ROMROB Mn^II^—O(H) bond length (mean 2.134 Å) when (**1**) is compared to ROMROB which has two additional protons to compensate for the two additional electrons. In ROMROB, the Mn^II^—O(phenolate) and Mn^II^—N(imine) bonds are also elongated (mean lengths 2.011 and 2.027 Å, respectively). (**1**) thus provides a rare structural example of variations in the metal coordination sphere to accommodate change in the metal oxidation state. The flexibility of the lattice, formed using the partly deprotonated H_4_ *L* ligand, permits distortion of the structure in the solid state to allow for changes in the charge and spin state of the Mn atom without disrupting the integrity of the crystal structure.

## Supra­molecular features   

**Figure 3:**
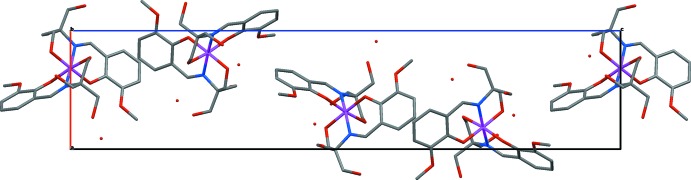
Crystal packing of (**1**) showing the helical arrangement of Mn^IV^(H_2_ *L*)_2_ mol­ecules in the *c*-axis direction. H atoms are not shown.

In the crystal lattice, individual [Mn^IV^(H_2_ *L*)_2_] mol­ecules are stacked in a helical fashion along the *c* axis, as shown in Fig. 3 ▸, with the minimum Mn⋯Mn distances inside a column being 10.28 Å. Mol­ecules that are translated by one unit cell in the *a*-axis direction [Mn⋯Mn distance equals the *a*-axial length, 8.0953 (2) Å] are inter­twined by inter­molecular hydrogen bonds between the hydroxyl groups and phenolic and meth­oxy oxygen atoms. There is also a possible hydrogen-bonding inter­action between one hydroxyl group (O113) and the solvent water mol­ecule (O1) considering the O113⋯O1 distance of 2.17 (2) but as the H atoms on O1 could not be located this contact could not be confirmed. Details of the hydrogen bonding are given in Table 2 ▸.

## Database survey   

A search of the Cambridge Structural Database (CSD Version 5.36 with one update; Groom & Allen, 2014 ▸) for metal complexes of this ligand reveals the crystal structures of above 30 compounds, mostly comprising polynuclear homo- Co^II^Co^III^, V_2_, Cu_4_, Mn_4_, Ni_4_, Ln_9_ and Ln_10_ and heterometallic 1*s*–3*d* and 3*d*–4*f* assemblies of 4–20 nuclearity. Mononuclear complexes of this ligand are limited to five Mn, Ni and Mo structures. The ligand mol­ecules exist in either doubly or triply deprotonated forms, adopt a chelating-bridging mode and form five- and six-membered rings. The H_4_ *L* ligand can stabilize manganese in various oxidation states. Apart from Mn^II^ (ROMROB) and Mn^IV^ [(**1**); IGOSII] complexes, the structure of the Mn^III^ derivative, [Mn_4_(H*L*)_2_(H_2_ *L*)_2_(CH_3_OH)_4_](ClO_4_)_2_]·4CH_3_OH has also been reported (Zhu *et al.*, 2014 ▸). Stabilization of Mn^IV^ species by similar ligands with phenolate oxygen atoms has been reported previously with details of three structures of [Mn^IV^N_2_O_4_] complexes with tridentate Schiff base ligands similar to H_4_ *L* (Kessissoglou *et al.*, 1987 ▸; Chandra *et al.*, 1990 ▸; Pradeep *et al.*, 2004 ▸).

## Synthesis and crystallization   

2-Hy­droxy-3-meth­oxy-benzaldehyde (0.30 g, 2 mmol) and tris­(hy­droxy­meth­yl)amino­methane (0.24 g, 2 mmol), were added to methanol (20 ml) and stirred magnetically for 30 min. Next zinc powder (0.07 g, 1 mmol) and MnCl_2_·4H_2_O (0.20 g, 1 mmol) were added to the yellow solution and the mixture was heated to 323 K under stirring until total dissolution of the zinc powder was observed (1 h). The resulting brown solution was filtered and allowed to stand at room temperature. Black microcrystals of the title compound were formed in several days. They were collected by filter-suction, washed with dry Pr^i^OH and finally dried *in vacuo* (yield: 43%).

The IR spectrum of powdered (**1**) in the range 4000–400 cm^−1^ shows all the characteristic Schiff base vibration bands: ν(OH), ν(CH) and ν(C=N) at 3400, 3000–2840, and 1602 cm^−1^, respectively (see Supplementary data). A strong peak at 1618 cm^−1^ is due to the bending of the H_2_O mol­ecule, providing evidence of the presence of water in (**1**). The major feature of the X-band solid-state EPR spectrum of (**1**) at 77 K is a strong and broad signal at *g* ∼4 and a weak but resolved response at *g* ∼2 (see Supplementary data). This corresponds to strong axial distortion with small zero-field splitting, *2D* >> *hυ* (*hυ* 0.31 cm^−1^ at the X-band frequency) in agreement with structural findings. The ^55^Mn hyperfine structure is not resolved.

## Refinement   

Crystal data, data collection and structure refinement details are summarized in Table 3 ▸. The solvent was modelled as a water mol­ecule with the site occupancy refined to 0.195 (15). Associated hydrogen atoms were not located. The OH hydrogen atoms H112 and H113 were refined using a riding model with *U*_iso_(H) = 1.5*U*_eq_(O). All hydrogen atoms bound to carbon were included in calculated positions and refined using a riding model with isotropic displacement parameters based on those of the parent atom [C—H = 0.95 Å, *U*_iso_(H) = 1.2*U*_eq_(C) for CH and CH_2_, 1.5*U*_eq_(C) for CH_3_].

## Supplementary Material

Crystal structure: contains datablock(s) I, global. DOI: 10.1107/S2056989015018551/sj5481sup1.cif

Structure factors: contains datablock(s) I. DOI: 10.1107/S2056989015018551/sj5481Isup2.hkl

Supporting information file. DOI: 10.1107/S2056989015018551/sj5481Isup3.pdf

Supporting information file. DOI: 10.1107/S2056989015018551/sj5481Isup4.tif

CCDC reference: 1429416

Additional supporting information:  crystallographic information; 3D view; checkCIF report

## Figures and Tables

**Table 1:** Selected geometric parameters (, )

Mn1O111	1.871(4)	Mn1N10	1.992(5)
Mn1O11	1.939(4)		
			
O111Mn1O111^i^	94.0(3)	O111^i^Mn1N10	88.07(19)
O111Mn1O11^i^	89.58(16)	O11^i^Mn1N10	98.44(18)
O111Mn1O11	172.89(18)	O11Mn1N10	89.82(17)
O11^i^Mn1O11	87.6(2)	N10Mn1N10^i^	168.6(3)
O111Mn1N10	84.14(18)		

Symmetry code: (i) 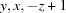.

**Table 2:** Hydrogen-bond geometry (, )

*D*H*A*	*D*H	H*A*	*D* *A*	*D*H*A*
O112H112O11^ii^	0.84	2.2	2.850(7)	134
O112H112O16^ii^	0.84	2.1	2.802(7)	141
O113H113O112^iii^	0.84	2.3	2.965(12)	137

Symmetry codes: (ii) 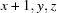; (iii) 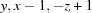.

**Table 3:** Experimental details

Crystal data	
Chemical formula	[Mn(C_12_H_15_NO_5_)_2_]0.39H_2_O
*M*_r_	568.46
Crystal system, space group	Tetragonal, *P*4_1_2_1_2
Temperature (K)	100
*a*, *c* ()	8.0953(2), 37.568(2)
*V* (^3^)	2461.97(18)
*Z*	4
Radiation type	Cu *K*
(mm^1^)	4.92
Crystal size (mm)	0.09 0.08 0.01

Data collection	
Diffractometer	Oxford Diffraction Gemini
Absorption correction	Analytical [*CrysAlis PRO* (Agilent, 2014 ▸) using an expression derived by Clark Reid (1995 ▸)]
*T*_min_, *T*_max_	0.695, 0.946
No. of measured, independent and observed [*I* > 2(*I*)] reflections	18553, 2214, 1885
*R*_int_	0.103
(sin /)_max_ (^1^)	0.600

Refinement	
*R*[*F* ^2^ > 2(*F* ^2^)], *wR*(*F* ^2^), *S*	0.052, 0.136, 1.05
No. of reflections	2214
No. of parameters	181
H-atom treatment	H-atom parameters constrained
_max_, _min_ (e ^3^)	0.54, 0.34
Absolute structure	Flack *x* determined using 584 quotients [(*I* ^+^)(*I* )]/[(*I* ^+^)+(*I* )] (Parsons *et al.*, 2013 ▸).
Absolute structure parameter	0.007(6)

Computer programs: *CrysAlis PRO* (Agilent, 2014 ▸), *SIR92* (Altomare *et al.*, 1993 ▸), *SHELXL2014* (Sheldrick, 2015 ▸), *DIAMOND* (Brandenburg, 1999 ▸) and *WinGX* (Farrugia, 2012 ▸).

## supplementary crystallographic information

### Crystal data

**Table:**

[Mn(C_12_H_15_NO_5_)_2_]·0.39H_2_O	*D*_x_ = 1.534 Mg m^−^^3^
*M**_r_* = 568.46	Cu *K*α radiation, λ = 1.54178 Å
Tetragonal, *P*4_1_2_1_2	Cell parameters from 2851 reflections
Hall symbol: P 4abw 2nw	θ = 3.5–67.6°
*a* = 8.0953 (2) Å	µ = 4.92 mm^−^^1^
*c* = 37.568 (2) Å	*T* = 100 K
*V* = 2461.97 (18) Å^3^	Plate, black
*Z* = 4	0.09 × 0.08 × 0.01 mm
*F*(000) = 1188	

### Data collection

**Table:**

Oxford Diffraction Gemini diffractometer	2214 independent reflections
Graphite monochromator	1885 reflections with *I* > 2σ(*I*)
Detector resolution: 10.4738 pixels mm^-1^	*R*_int_ = 0.103
ω scans	θ_max_ = 67.7°, θ_min_ = 4.7°
Absorption correction: analytical [*CrysAlis PRO* (Agilent, 2014) using an expression derived by Clark & Reid (1995)]	*h* = −9→9
*T*_min_ = 0.695, *T*_max_ = 0.946	*k* = −6→9
18553 measured reflections	*l* = −42→44

### Refinement

**Table:**

Refinement on *F*^2^	Hydrogen site location: inferred from neighbouring sites
Least-squares matrix: full	H-atom parameters constrained
*R*[*F*^2^ > 2σ(*F*^2^)] = 0.052	*w* = 1/[σ^2^(*F*_o_^2^) + (0.0691*P*)^2^ + 1.3653*P*] where *P* = (*F*_o_^2^ + 2*F*_c_^2^)/3
*wR*(*F*^2^) = 0.136	(Δ/σ)_max_ < 0.001
*S* = 1.05	Δρ_max_ = 0.54 e Å^−^^3^
2214 reflections	Δρ_min_ = −0.34 e Å^−^^3^
181 parameters	Absolute structure: Flack *x* determined using 584 quotients [(*I*^+^)-(*I*^-^)]/[(*I*^+^)+(*I*^-^)] (Parsons *et al*., 2013).
0 restraints	Absolute structure parameter: −0.007 (6)

### Special details

**Table:**

Geometry. All e.s.d.'s (except the e.s.d. in the dihedral angle between two l.s. planes) are estimated using the full covariance matrix. The cell e.s.d.'s are taken into account individually in the estimation of e.s.d.'s in distances, angles and torsion angles; correlations between e.s.d.'s in cell parameters are only used when they are defined by crystal symmetry. An approximate (isotropic) treatment of cell e.s.d.'s is used for estimating e.s.d.'s involving l.s. planes.
Refinement. The solvent was modelled as a water molecule with a site occupancy refined to 0.195 (15). Associated hydrogen atoms were not located.

### Fractional atomic coordinates and isotropic or equivalent isotropic displacement parameters (Å^2^)

**Table:**

	*x*	*y*	*z*	*U*_iso_*/*U*_eq_	Occ. (<1)
Mn1	0.68511 (10)	0.68511 (10)	0.5	0.0286 (3)	
C11	0.6187 (6)	0.4968 (6)	0.56423 (15)	0.0308 (12)	
O11	0.5567 (4)	0.5690 (5)	0.53567 (9)	0.0333 (8)	
C12	0.7784 (6)	0.4298 (7)	0.56576 (14)	0.0330 (13)	
C13	0.8302 (7)	0.3446 (7)	0.59647 (14)	0.0365 (13)	
H13	0.9372	0.2963	0.5969	0.044*	
C14	0.7305 (7)	0.3297 (7)	0.62568 (16)	0.0385 (13)	
H14	0.7677	0.2721	0.6462	0.046*	
C15	0.5729 (7)	0.4005 (7)	0.62492 (15)	0.0394 (13)	
H15	0.5037	0.393	0.6453	0.047*	
C16	0.5167 (7)	0.4815 (7)	0.59479 (14)	0.0329 (11)	
O16	0.3648 (5)	0.5520 (5)	0.59131 (11)	0.0414 (10)	
C161	0.2522 (8)	0.5349 (8)	0.62010 (18)	0.0443 (15)	
H16A	0.2355	0.4174	0.6253	0.066*	
H16B	0.1462	0.5854	0.6137	0.066*	
H16C	0.2971	0.5901	0.6412	0.066*	
C10	0.8942 (7)	0.4456 (7)	0.53655 (15)	0.0364 (13)	
H10	0.9951	0.3863	0.5385	0.044*	
N10	0.8733 (5)	0.5316 (6)	0.50849 (12)	0.0349 (11)	
C101	1.0070 (8)	0.5533 (9)	0.48146 (17)	0.0502 (17)	
C111	0.9865 (8)	0.7342 (9)	0.46941 (19)	0.0508 (17)	
H11A	1.0338	0.8087	0.4876	0.061*	
H11B	1.0475	0.7518	0.4469	0.061*	
O111	0.8214 (5)	0.7719 (5)	0.46438 (11)	0.0444 (10)	
C112	1.1779 (9)	0.5228 (11)	0.4957 (2)	0.072 (2)	
H11C	1.1897	0.4044	0.5018	0.087*	
H11D	1.26	0.5492	0.477	0.087*	
O112	1.2110 (7)	0.6187 (10)	0.52604 (19)	0.096 (2)	
H112	1.2848	0.5727	0.5382	0.143*	
C113	0.9654 (11)	0.4419 (9)	0.44961 (19)	0.065 (2)	
H11E	0.8528	0.4678	0.441	0.078*	
H11F	1.044	0.464	0.43	0.078*	
O113	0.9740 (11)	0.2689 (7)	0.45943 (17)	0.105 (3)	
H113	0.9002	0.2478	0.4745	0.157*	
O1	0.787 (3)	0.090 (3)	0.4441 (6)	0.048 (9)	0.195 (15)

### Atomic displacement parameters (Å^2^)

**Table:**

	*U*^11^	*U*^22^	*U*^33^	*U*^12^	*U*^13^	*U*^23^
Mn1	0.0282 (4)	0.0282 (4)	0.0293 (6)	−0.0008 (5)	−0.0022 (4)	0.0022 (4)
C11	0.031 (3)	0.025 (3)	0.036 (3)	−0.002 (2)	−0.005 (2)	0.000 (2)
O11	0.026 (2)	0.039 (2)	0.034 (2)	−0.0011 (15)	−0.0036 (16)	0.0023 (17)
C12	0.034 (3)	0.030 (3)	0.035 (3)	−0.005 (2)	−0.003 (2)	0.002 (2)
C13	0.031 (3)	0.037 (3)	0.042 (3)	−0.001 (2)	−0.006 (2)	0.003 (2)
C14	0.043 (3)	0.035 (3)	0.037 (3)	−0.005 (3)	−0.007 (2)	0.008 (2)
C15	0.046 (3)	0.035 (3)	0.037 (3)	−0.008 (3)	0.002 (3)	0.001 (2)
C16	0.032 (3)	0.029 (3)	0.037 (3)	−0.005 (2)	0.001 (2)	0.003 (2)
O16	0.032 (2)	0.052 (2)	0.041 (2)	0.0051 (17)	0.0082 (17)	0.0053 (19)
C161	0.038 (3)	0.043 (4)	0.052 (4)	0.002 (3)	0.011 (3)	0.007 (3)
C10	0.026 (3)	0.041 (3)	0.042 (3)	0.002 (2)	−0.002 (2)	0.000 (3)
N10	0.028 (2)	0.045 (3)	0.032 (2)	−0.002 (2)	−0.0006 (18)	0.007 (2)
C101	0.036 (3)	0.067 (5)	0.047 (4)	0.008 (3)	0.007 (3)	0.012 (3)
C111	0.045 (4)	0.060 (4)	0.047 (4)	−0.008 (3)	0.004 (3)	0.016 (3)
O111	0.043 (2)	0.048 (2)	0.041 (2)	−0.010 (2)	−0.0092 (19)	0.0110 (18)
C112	0.039 (4)	0.098 (6)	0.080 (6)	0.006 (4)	0.019 (4)	0.035 (5)
O112	0.049 (3)	0.142 (6)	0.096 (5)	−0.025 (4)	−0.025 (3)	0.055 (4)
C113	0.085 (6)	0.056 (4)	0.055 (4)	0.015 (4)	0.024 (4)	0.012 (4)
O113	0.184 (8)	0.053 (3)	0.077 (4)	0.048 (4)	0.067 (5)	0.025 (3)
O1	0.055 (16)	0.037 (13)	0.052 (15)	0.017 (11)	0.016 (11)	0.008 (10)

### Geometric parameters (Å, º)

**Table:**

Mn1—O111	1.871 (4)	C161—H16B	0.98
Mn1—O111^i^	1.871 (4)	C161—H16C	0.98
Mn1—O11^i^	1.939 (4)	C10—N10	1.275 (7)
Mn1—O11	1.939 (4)	C10—H10	0.95
Mn1—N10	1.992 (5)	N10—C101	1.495 (7)
Mn1—N10^i^	1.992 (5)	C101—C112	1.504 (10)
C11—O11	1.321 (6)	C101—C113	1.536 (10)
C11—C12	1.403 (8)	C101—C111	1.542 (10)
C11—C16	1.420 (8)	C111—O111	1.384 (8)
C12—C13	1.408 (7)	C111—H11A	0.99
C12—C10	1.449 (8)	C111—H11B	0.99
C13—C14	1.368 (8)	C112—O112	1.405 (11)
C13—H13	0.95	C112—H11C	0.99
C14—C15	1.399 (9)	C112—H11D	0.99
C14—H14	0.95	O112—H112	0.84
C15—C16	1.384 (8)	C113—O113	1.450 (9)
C15—H15	0.95	C113—H11E	0.99
C16—O16	1.362 (7)	C113—H11F	0.99
O16—C161	1.421 (7)	O113—H113	0.84
C161—H16A	0.98		
			
O111—Mn1—O111^i^	94.0 (3)	H16A—C161—H16B	109.5
O111—Mn1—O11^i^	89.58 (16)	O16—C161—H16C	109.5
O111^i^—Mn1—O11^i^	172.89 (19)	H16A—C161—H16C	109.5
O111—Mn1—O11	172.89 (18)	H16B—C161—H16C	109.5
O111^i^—Mn1—O11	89.58 (16)	N10—C10—C12	126.0 (5)
O11^i^—Mn1—O11	87.6 (2)	N10—C10—H10	117
O111—Mn1—N10	84.14 (18)	C12—C10—H10	117
O111^i^—Mn1—N10	88.07 (19)	C10—N10—C101	122.0 (5)
O11^i^—Mn1—N10	98.44 (18)	C10—N10—Mn1	125.1 (4)
O11—Mn1—N10	89.82 (17)	C101—N10—Mn1	111.8 (4)
O111—Mn1—N10^i^	88.07 (19)	N10—C101—C112	113.9 (5)
O111^i^—Mn1—N10^i^	84.14 (18)	N10—C101—C113	107.5 (6)
O11^i^—Mn1—N10^i^	89.82 (17)	C112—C101—C113	112.5 (7)
O11—Mn1—N10^i^	98.44 (18)	N10—C101—C111	103.5 (5)
N10—Mn1—N10^i^	168.6 (3)	C112—C101—C111	111.1 (6)
O11—C11—C12	123.7 (5)	C113—C101—C111	107.8 (5)
O11—C11—C16	118.3 (5)	O111—C111—C101	110.7 (5)
C12—C11—C16	118.0 (5)	O111—C111—H11A	109.5
C11—O11—Mn1	124.9 (3)	C101—C111—H11A	109.5
C11—C12—C13	119.8 (5)	O111—C111—H11B	109.5
C11—C12—C10	122.1 (5)	C101—C111—H11B	109.5
C13—C12—C10	118.1 (5)	H11A—C111—H11B	108.1
C14—C13—C12	121.6 (5)	C111—O111—Mn1	112.9 (4)
C14—C13—H13	119.2	O112—C112—C101	111.9 (7)
C12—C13—H13	119.2	O112—C112—H11C	109.2
C13—C14—C15	119.0 (5)	C101—C112—H11C	109.2
C13—C14—H14	120.5	O112—C112—H11D	109.2
C15—C14—H14	120.5	C101—C112—H11D	109.2
C16—C15—C14	120.7 (5)	H11C—C112—H11D	107.9
C16—C15—H15	119.7	C112—O112—H112	109.5
C14—C15—H15	119.7	O113—C113—C101	111.0 (6)
O16—C16—C15	125.0 (5)	O113—C113—H11E	109.4
O16—C16—C11	114.3 (5)	C101—C113—H11E	109.4
C15—C16—C11	120.7 (5)	O113—C113—H11F	109.4
C16—O16—C161	117.7 (5)	C101—C113—H11F	109.4
O16—C161—H16A	109.5	H11E—C113—H11F	108
O16—C161—H16B	109.5	C113—O113—H113	109.5

Symmetry code: (i) *y*, *x*, −*z*+1.

### Hydrogen-bond geometry (Å, º)

**Table:**

*D*—H···*A*	*D*—H	H···*A*	*D*···*A*	*D*—H···*A*
O112—H112···O11^ii^	0.84	2.2	2.850 (7)	134
O112—H112···O16^ii^	0.84	2.1	2.802 (7)	141
O113—H113···O112^iii^	0.84	2.3	2.965 (12)	137

Symmetry codes: (ii) *x*+1, *y*, *z*; (iii) *y*, *x*−1, −*z*+1.
