# Supplementary figures and images for: Crystal structure of bis­(2-{[1,1-bis­(hy­droxy­meth­yl)-2-oxidoeth­yl]imino­meth­yl}-6-meth­oxy­phenolato)manganese(IV) 0.39-hydrate
Source: Acta Crystallogr E Crystallogr Commun. 2015 Oct 10;71(Pt 11):1307–10. doi: 10.1107/S2056989015018551 (PMC4645066; doi:10.1107/S2056989015018551)

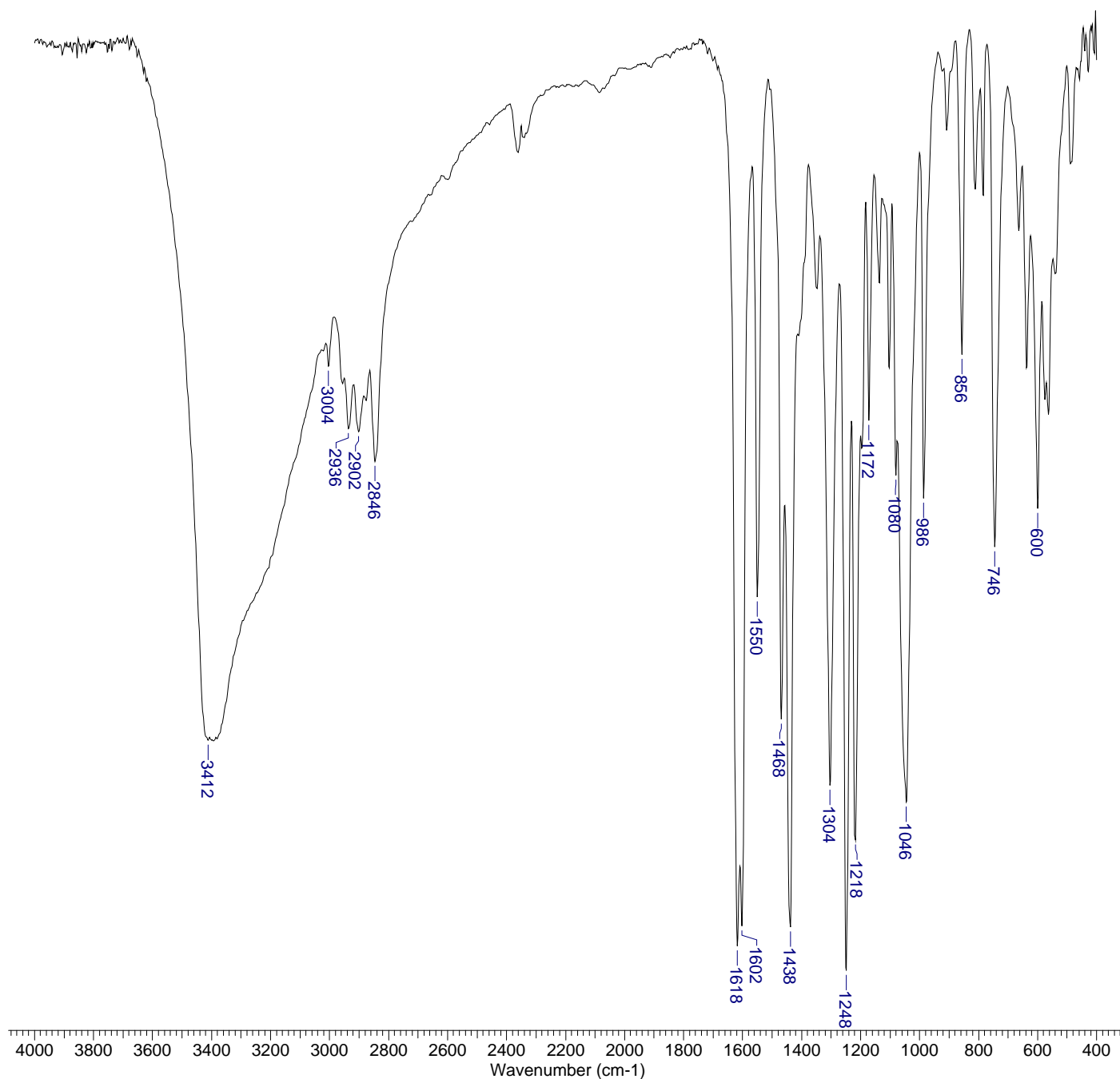

| No | cm-1    | %T     | Intensity |
|----|---------|--------|-----------|
| 1  | 600.00  | 42.577 | M         |
| 2  | 746.00  | 39.307 | M         |
| 3  | 856.00  | 55.851 | M         |
| 4  | 986.00  | 43.480 | M         |
| 5  | 1046.00 | 17.293 | S         |
| 6  | 1080.00 | 45.466 | M         |
| 7  | 1172.00 | 50.165 | M         |
| 8  | 1218.00 | 14.049 | S         |
| 9  | 1248.00 | 2.788  | VS        |
| 10 | 1304.00 | 18.771 | S         |
| 11 | 1438.00 | 6.529  | VS        |
| 12 | 1468.00 | 24.451 | S         |
| 13 | 1550.00 | 34.955 | S         |
| 14 | 1602.00 | 6.639  | VS        |
| 15 | 1618.00 | 4.925  | VS        |
| 16 | 2846.00 | 46.634 | M         |
| 17 | 2902.00 | 49.206 | M         |
| 18 | 2936.00 | 49.483 | M         |
| 19 | 3004.00 | 54.842 | M         |
| 20 | 3412.00 | 22.628 | S         |

Supplement: Supplementary file 3 [file e-71-01307-Isup3.pdf]

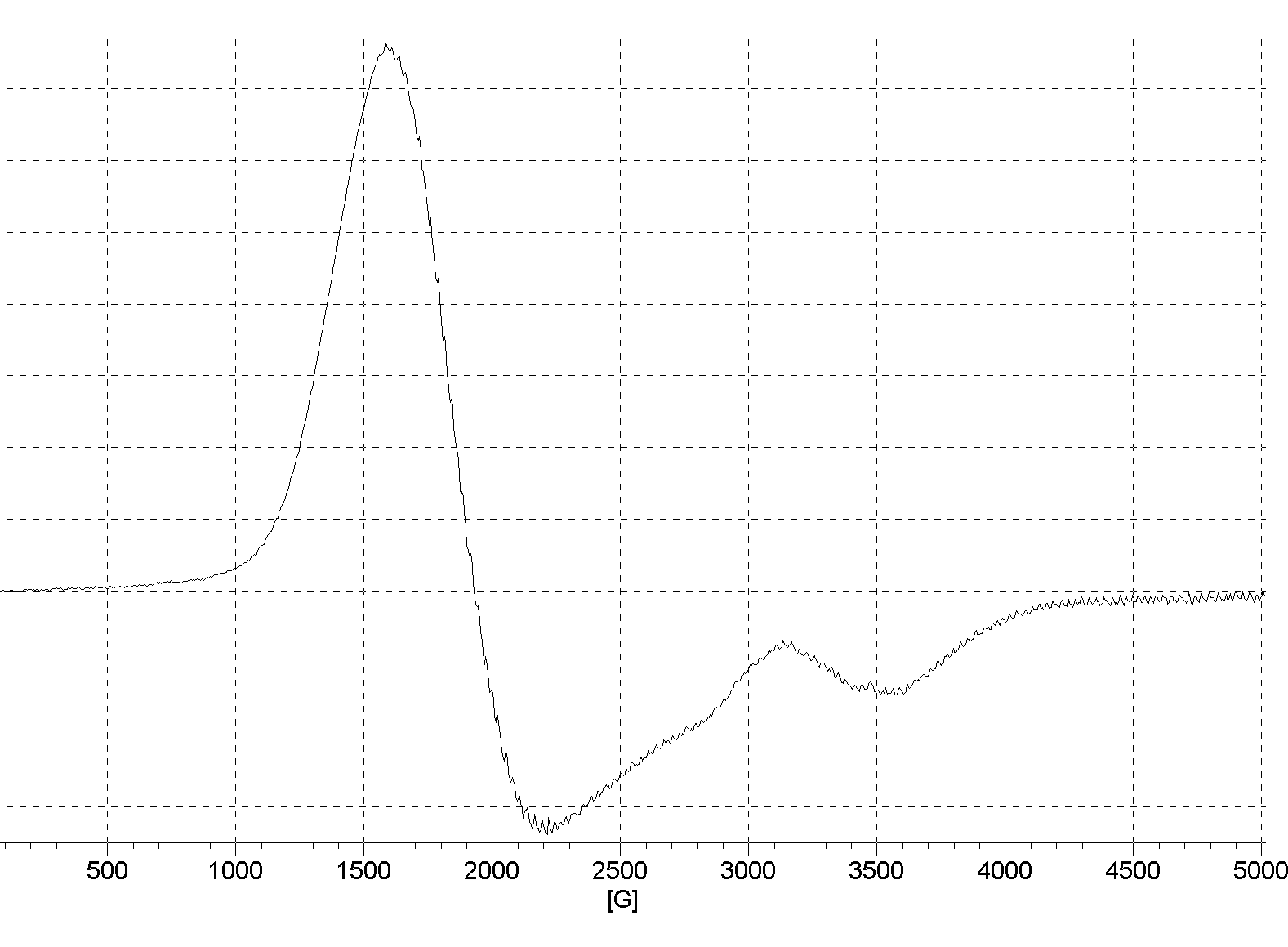

Supplement: Supplementary file 4 [file e-71-01307-Isup4.tif]
